# Predicting dyslexia using prereading skills: the role of sensorimotor and cognitive abilities

**DOI:** 10.1111/jcpp.12488

**Published:** 2015-12-12

**Authors:** Julia M. Carroll, Jonathan Solity, Laura R. Shapiro

**Affiliations:** ^1^ Centre for Research in PsychologyBehaviour and AchievementCoventry UniversityCoventryUK; ^2^ Optima PsychologyLeamington SpaUK; ^3^ School of Health and Life SciencesAston UniversityBirminghamUK

**Keywords:** Dyslexia, educational attainment, longitudinal studies, prediction, phonological processing

## Abstract

**Background:**

It is well established that phonological awareness, print knowledge and rapid naming predict later reading difficulties. However, additional auditory, visual and motor difficulties have also been observed in dyslexic children. It is examined to what extent these difficulties can be used to predict later literacy difficulties.

**Method:**

An unselected sample of 267 children at school entry completed a wide battery of tasks associated with dyslexia. Their reading was tested 2, 3 and 4 years later and poor readers were identified (*n* = 42). Logistic regression and multiple case study approaches were used to examine the predictive validity of different tasks.

**Results:**

As expected, print knowledge, verbal short‐term memory, phonological awareness and rapid naming were good predictors of later poor reading. Deficits in visual search and in auditory processing were also present in a large minority of the poor readers. Almost all poor readers showed deficits in at least one area at school entry, but there was no single deficit that characterised the majority of poor readers.

**Conclusions:**

Results are in line with Pennington's (2006) multiple deficits view of dyslexia. They indicate that the causes of poor reading outcome are multiple, interacting and probabilistic, rather than deterministic.

## Introduction

Prospective studies of developmental dyslexia are useful for at least two reasons. The first is a practical one: they can help to establish which measures would be useful screening tools to predict future difficulties. This could help select children who should be given early support or intervention to try to avoid a negative cycle of attainment (Stanovich, 1986). The second way in which they can be useful is in shedding light on theoretical views of the possible causes of reading difficulties. This paper aims to address both of these issues.

Several studies have shown a consistent set of predictors of reading difficulties, including phonological awareness (PA), letter and print knowledge (PK), and rapid naming (Muter, Hulme, Snowling, & Taylor, 1998; Torgesen, Wagner, Rashotte, Burgess, & Hecht, 1997; Wagner et al., 1997). However, it is less certain whether these measures accurately predict which children will show later reading difficulties. Elbro, Borstrom, and Peterson (1998) achieved a prediction rate of between 84% and 79% in a sample of children at family risk of dyslexia, depending on the cut‐off value used. Their predictors included letter knowledge and multiple measures of phonological processing.

Puolakanaho et al. (2007) suggest that a sensitivity of 90% (e.g. that 90% of ‘true’ dyslexics would be correctly predicted) is acceptable for clinical use. However, in their study this resulted in low specificity (65.8%), meaning that over a third of their typically developing children would be misclassified as having dyslexia. While this approach would be effective in selecting children at risk, it would result in a large number of children receiving potentially unnecessary intervention. This classification also relies on knowledge of the history of dyslexia in the family, something that is often not available when screening classes of children. Puolakanaho et al. (2007) carried out their classification based on rapid naming, PA and letter knowledge between 3.5 and 5.5 years of age.

The present study builds on this research in several ways. First, it includes children across the full range of ability, thus providing a much more ecologically valid screening sample than a set of children with a family history of dyslexia and matched typically developing controls. Previous studies have excluded some borderline cases (e.g. Le Jan et al., 2011), or used samples that are preselected in some way (e.g. on the basis of parental dyslexia). These samples typically exclude children with other developmental difficulties from the control group. Second, it focuses only on prereading children, thus minimising the possible reciprocal effect of reading on related cognitive skills such as phoneme awareness (Castles & Coltheart, 2004; Morais, Cary, Alegria, & Bertelson, 1979). Finally and perhaps most crucially, it includes a wide range of measures, including measures of overall cognitive ability, sensory processing and motor skills. It is well established that at least some children with dyslexia show deficits in these areas. Although classical theories of dyslexia highlight deficits in phonological processing and PA as the major cause (e.g. Snowling, 2000; Stanovich, 1988), there remains much debate in the field.

Pennington and colleagues have argued that dyslexia, in line with other developmental disorders, occurs as a result of a combination of deficits in multiple areas (Pennington, 2006; Pennington et al., 2012). Although evidence for the phonological deficit comes from many different experimental designs, including longitudinal and intervention studies, evidence for other deficits in dyslexia tends to come from group difference studies in which diagnosed dyslexics are compared to typical controls. These studies do not allow assessment of whether a given deficit plays a causal role in a disorder and of what proportion of dyslexic children show the deficit (see White et al., 2006 for more detailed discussion of this issue).

The range of nonphonological deficits observed in dyslexic participants fits within a multiple‐deficit view. For example, many researchers have demonstrated group deficits in auditory processing in children with dyslexia and preschool children who go on to be dyslexic (Boets et al., 2011; Hamalainen et al., 2013), though others have argued that these deficits are not causally linked to reading and spelling (e.g. Marshall, Snowling, & Bailey, 2001; McArthur, Ellis, Atkinson, & Coltheart, 2008). Plakas, van Zuijen, van Leeuwen, Thomson, and van der Leij (2013) found that while children with a genetic risk of dyslexia were impaired on an auditory processing task (amplitude rise time), poor readers were not significantly worse on the task than high risk good readers. They argue that auditory processing forms one of a complex set of interacting causal factors.

Similarly, difficulties with motor and balance skills have been associated with dyslexia (e.g. Nicolson, Fawcett, & Dean, 2001). Nicolson and Fawcett (1990) argued that a cerebellar dysfunction causes dyslexic participants to have difficulties in all types of automatic learned tasks (including reading and motor tasks), though more recent versions of this theory focus more specifically on procedural learning difficulties (Nicolson, Fawcett, Brooks, & Needle, 2010).

There is also evidence that subtle visual difficulties could cause reading difficulties. For example, Bosse, Tainturier, and Valdois (2007) found both phonological and visual attention deficits in dyslexic children. Examination of scatterplots indicated that children with dyslexia tended to exhibit either a visual deficit or a phonological deficit in comparison to their typically developing peers, providing more evidence in favour of the idea of multiple causes of dyslexia.

Some recent research has given positive evidence for including sensory and motor skills in the prediction of dyslexia. Le Jan et al. (2011) found that a combination of auditory, visuo‐attentional, phonological and morphological measures provided a highly effective screening measure with 94% of children correctly classified (though in this study nondyslexic weak readers were excluded, which may have increased the screen's effectiveness).

One published measure that includes a broader range of skills is the Dyslexia Early Screening Test (DEST; Nicolson & Fawcett, 1996). This test purports to predict dyslexia in individuals using ten short tests of rapid naming, PA, auditory processing, letter knowledge, short‐term memory, motor skills and balance. However, an evaluation of the DEST (Simpson & Everatt, 2005) suggests that the test as a whole has limited predictive power, and that two subtests (rapid naming and auditory processing), together with measures of letter knowledge, provided the best prediction overall. Averaging over these measures and sensorimotor measures, as suggested in the DEST, actually reduced the predictive power of the variables. The present study addresses this issue by using a combination of measures taken from the DEST and additional measures of cognitive and sensori‐motor processing.

While studies that examine group differences can demonstrate an average deficit across the group, they may mask individual differences in patterns of deficit. For example, if 50% of dyslexic individuals had a deficit in PA while the remaining 50% did not, PA would likely still show a group level deficit, even though it does not explain the literacy difficulties of 50% of the sample. In order to focus more on individual patterns of attainment, some researchers have turned to a multiple case study approach. For example, Ramus et al. (2003) examined patterns of deficits in 16 dyslexic university students. While a significant minority of the group showed deficits in visual, motor and auditory processing, the only deficit that was universal was a deficit in phonological processing. The authors thus concluded that phonological processing was causally implicated in dyslexia, while the other deficits were epiphenomenal.

However, a replication with school‐aged dyslexic children (White et al., 2006) did not show such a consistent pattern: only around half of the sample of dyslexic children showed deficits in PA with smaller, but still significant, proportions of the sample showing sensorimotor deficits. There are three key possible reasons for this discrepancy between adult and child samples. First, PA deficits may become more acute with increasing age, because of the reciprocal influence of reading on PA. Second, the remediation received by the dyslexic school children may have temporarily ameliorated their deficits, which then reappear in university when this support typically ceases. Third, it could be that dyslexic university students are unusual in their profiles: to attend university despite dyslexic difficulties suggests a particular profile of strengths and weaknesses, including high IQ and motivation. One way to examine these possible explanations is to use a prospective study design to assess the deficits that were present in poor readers before they began reading instruction. If only a minority of poor readers show PA deficits prior to the onset of schooling, this provides evidence in favour of the first explanation.

Pennington et al. (2012) compared different theoretical explanations for dyslexic difficulties at an individual level. They found that approximately one‐third of their sample of dyslexic children fit best with a multiple deficits model, while 26% fit a single deficit model, leaving 40% not fitting either model perfectly. Most of these additional cases showed deficits in more than one area, but their best fitting regression model was a single deficit model, possibly supporting Ramus et al.'s (2003) epiphenomenal view. Nonetheless, these data suggest that deterministic, single cause models of dyslexia cannot explain all cases, and that we should think of these causes as probabilistic.

There has been a long‐term discussion on the role of IQ in dyslexia. Classically, dyslexia was defined in terms of reading difficulties that are significantly below a child's IQ, and dyslexic readers were contrasted with so‐called ‘garden variety’ poor readers (Stanovich, 1988). It is well established that there is a significant association between reading level and nonverbal IQ (Swanson, Trainin, Necoechea, & Hammill, 2003). However, many researchers have argued that there is no qualitative difference between the difficulties shown by these two groups (Share & Shalev, 2004; Stanovich & Siegel, 1994). The current study also provides an opportunity to examine whether the predictors of dyslexia vary depending on whether the dyslexia is defined in terms of only poor reading or whether it is defined in terms of a discrepancy between reading and IQ.

This study examines the following questions in an unselected sample of prereaders.

1. Are children who go on to become poor readers generally disadvantaged on cognitive and sensorimotor skills assessed prior to reading tuition?
2. How effectively do these measures predict which children will become poor readers, and which measures are important in the prediction?
3. What proportion of the sample show weaknesses in each area?
4. Is there evidence for multiple deficits causing dyslexia?
5. How do weaknesses in the different areas overlap?

## Method

### Participants

We collected data from four cohorts of children beginning reception classes (mean age 4 years, 6 months) in three primary schools in a large town in Worcestershire, United Kingdom. The schools had intakes of predominantly white British pupils of lower than average socioeconomic status, who began school with slightly below average attainments. Children in all schools were taught using a broad reading programme which included PA, phonics (decoding strategies) and recognition of high frequency words by sight (consistent with recommendations by Rose, 2006).

All parents of reception class children received an information sheet and opt‐out consent form prior to the start of the testing. Eleven of the 455 children registered in the reception classes were excluded from the study (due to assessment of learning difficulties, English as an additional language, reluctance to take part or parental opt‐out). One of the schools opted not to continue with the research after we had completed baseline assessments with the final cohort of children, reducing our sample size for the follow‐ups by 102. In addition, 75 further children dropped out of the study because they moved to different schools. In total, we collected data from 444 children at baseline, and 267 children took part in at least one follow‐up assessment, 260 at end of Year 1 (age 6 years 2 m), 184 at end of Year 2 (7 years 2 m) and 127 at end of Year 3 (8 years 2 m). Some of these children opted out of some tests, so the *n* for each measure fluctuates slightly (see Shapiro, Carroll, & Solity, 2013). In order to assess whether these 267 children differed from the original sample of 444, one‐way ANOVAs were calculated comparing the two groups on the initial background measures. There were no significant differences (*p* > .08) on any measure except verbal STM (*F*(1,418) = 11.54, *p* = .001) and balance (*F*(1,400) = 12.27, *p* < .001), in which the children who left the study slightly outperformed those remaining in the study. The remaining children can therefore be considered broadly representative of the sample.

### Design

Children were tested individually in their schools at five time‐points, baseline and four follow‐ups. Reading related skills were measured at baseline, early in the first term of formal schooling (4; 6 years; see Shapiro et al., 2013 for full details). The follow‐up tests of reading outcomes were conducted in the final half of the summer term, in one or two sessions of up to 20 min duration (with word reading and letter sound tasks administered prior to nonword reading tasks).

### Assessments

#### Measures taken at baseline

More detail is provided on these tasks in Shapiro et al. (2013). They are summarised below. Sample‐specific reliabilities for each measure are included in parentheses following the heading.

*Baseline print knowledge*. Letter sound knowledge was measured by presenting the child with a list of all 26 letters and asking the child to give the sound (Cronbach's *α *= .92). In the United Kingdom, children are taught the most frequent sound associated with each letter in the first year of school, before learning letter names. Sight word reading (hereafter sight words) was assessed using a list of the 100 most frequent words in written English. All children were asked to attempt the first 16 words, and after this, the test was terminated after five consecutive errors; the child's score was the total number of words read correctly (*α *= .94). Since children may learn to recognise digits before words, we also administered the digit‐naming test from the Dyslexia Early Screening Test (DEST; Nicolson & Fawcett, 1996). The child's score was the total number of digits identified correctly, out of seven test items (*α *= .89).

*Phonological awareness*. PA was measured using a range of standardised tests: the Rhyme Detection test from the Phonological Abilities Test (Muter, Hulme, & Snowling, 1997; *α *= .82); the DEST Rhyme Detection task (*α *= .40); the DEST phonological discrimination task (*α *= .44) and the DEST first letter sound test (*α *= .93). Substantial floor effects and a low reliability on the DEST Rhyme Detection task meant that it was not analysed further.

*Rapid automatised naming*. The DEST rapid picture‐naming test (Nicolson & Fawcett, 1996) was used which children are required to name a series of familiar objects as fast as possible (*α *= .75).

*Verbal short‐term memory*. VSTM was measured with two tasks: the Digit Span task from the DEST (*α *= .69) and a nonword repetition task created for this study (*α *= .71). This task was created using the 20 nonwords included in the Phonological Assessment Battery Nonword Reading task, and it included 10 monosyllabic and 10 bisyllabic words, many with consonant clusters.

*Speech production*. We used the speech rate test from the Phonological Abilities Test (PAT; Muter et al., 1997) to measure speed of speech production (*α *= .88). Children were asked to repeat a multisyllabic word (‘buttercup’) ten times, and total number of seconds to do this was recorded.

*Auditory processing*. Two tasks were used to measure children's auditory processing. Firstly, the DEST Sound Order test (*α *= .69), and secondly, an auditory processing task based on Tallal's (1980) task. A large number of participants (151, or 36%) did not successfully complete the training phase of the second task and so it is not reported further.

*Motor and balance*. Motor skill was measured using three standardised tasks: Shape Copying and Bead Threading from the DEST (published test–retest reliabilities *α *= .81 and *α *= .72 respectively), and a pegboard task (Annett, 2002). We also measured children's balance with the DEST Postural Stability task.

*Visual attention*. We based our visual attention task on the conjunction search task designed by Gerhardstein and Rovee‐Collier (2002) for use with very young children. The children were shown a display with differing numbers of ‘distractor’ dinosaurs and were asked to press a button indicating when a target dinosaur was present.

*Vocabulary and nonverbal reasoning*. The British Picture Vocabulary scale (BPVS; Dunn, Dunn, Whetton, & Burley, 1997), was used as a measure of receptive vocabulary (*α *= .83). Nonverbal reasoning was measured using Raven's Colored Progressive matrices, pasted onto wooden blocks (Raven, Raven, & Court, 1993; *α *= .79).

*Word reading accuracy*. The children completed the British Abilities Scale (BAS; Elliott, Murray, & Pearson, 1983) single word reading test at each time point.

#### Defining word reading difficulties

Two hundred and sixty‐seven of the 444 children originally tested completed the word reading measure at least once in years 1, 2 and 3. Raw scores were residualised for age at each time point and averaged across time‐points. In order to maximise the sample size, missing data was allowed, meaning this average score could be based on one, two or three time‐points. Word reading difficulties were then defined in two different ways. First, children with a mean score more than one standard deviation below the group mean were designated as poor readers (i.e. ‘below average’ according to most standardised tests). This procedure resulted in 18.0% of the sample being designated poor readers (PR: 47/262). Next, in order to select those children with a reading score significantly below what would be predicted given nonverbal ability, regression analysis was carried out predicting word reading with Ravens Matrices score (both controlled for age). The standardised residuals were saved and those with a standardised residual of >−1 (i.e. those with a reading score more than one *SD* below what would be predicted given nonverbal IQ) were classified as discrepancy‐defined poor readers (DD). This procedure resulted in 15% of children being classified as DD (40/262). Thirty‐four children were in both poor reader groups, indicating a high level of overlap between groups. In the following analyses, the PR classification group is used, and a note is included if the results differ with the DD classification group.

#### Composite variables and defining weaknesses on the predictor tasks

A series of composite variables was created which parallels planned theoretical constructs. In each case the variables were entered into an exploratory factor analysis which was forced to extract a single factor, which was then used in all the following analyses. In all cases the individual factor loadings were >.5, indicating that the composites were appropriate. Details of these composite variables are shown in Table 1. Seven further variables are retained as exogenous variables: DEST postural stability; Raven's nonverbal reasoning, vocabulary, DEST rapid naming, DEST sound order, speech rate and visual search.

**Table 1:** Factor loadings for the composite variables

Factor	Variables	Factor loadings	% variance explained
Print knowledge	Letter sound knowledge	.839	54.7%
	Sight word reading	.509	
	DEST digit naming	.824	
Phonological awareness	PAT rhyme	.728	51.8%
	DEST phoneme discrimination	.641	
	DEST 1^st^ letter (phoneme isolation)	.783	
VSTM	DEST digit span	.812	66.0%
	Nonword repetition	.812	
Motor	DEST shape copying	−.638	53.2%
	DEST bead threading	−.585	
	Pegboard left hand	.811	
	Pegboard right hand	.849	

On each of the eleven indicator variables and factors, the measure was residualised for age, then the lowest 16% of the group were designated as having a significant weakness in that area (equivalent to one SD below the mean for a normal distribution). This approach was used rather than selecting children on the basis of standard deviations, because not all of the measures were normally distributed.

## Results

### Mean group differences

**Figure 1:**
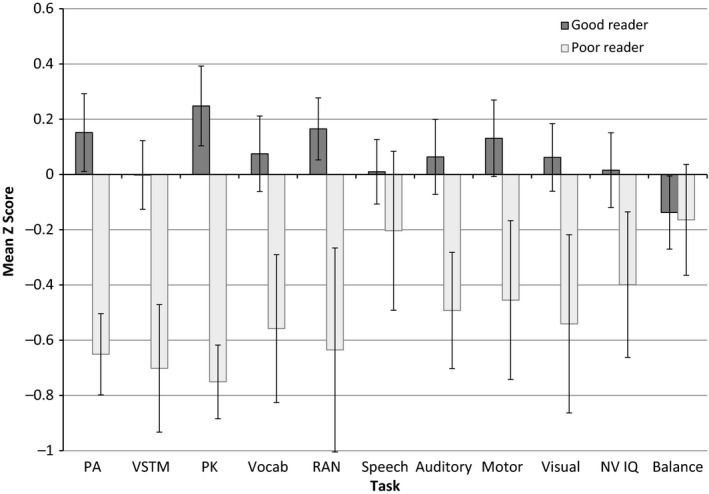
Mean group differences on each predictor variable

Mean group differences on each of the indicator variables after residualising for age are shown in Figure 1. The PR showed significantly poorer performance than good readers in each area, except postural stability and speech rate (PA: *t*(139.60) = 7.73, *p* < .001; VSTM: *t*(257) = 4.81, *p* < .001; motor skills: *t*(258) = −3.52, *p* = .001; PK: *t*(167.21) = 9.97, *p* < .001; rapid naming: *t*(53.61) = −5.30, *p* < .001; vocabulary: *t*(263) = 3.92, *p* < .001; sound order: *t*(88.93) = 4.35, *p* < .001; nonverbal IQ: *t*(260) = 2.59, *p* = .01; visual search: *t*(59.92) = −3.43, *p* = .001; postural stability: *t*(239) = 0.18, ns; speech rate: *t*(244) = −1.48, ns). This pattern largely replicates the group differences found in previous research and confirms the validity of selecting these tasks to predict literacy difficulties.^1^

### Which prereading skills are important in predicting which children will become poor readers?

A logistic regression analysis was carried out to predict outcome reading group. On the first step, school, age and sight word reading score at school entry were entered. ‘School’, in this context, means which of the three different schools the child attended. Only this predictor explained significant variance, showing that some variation is due to school or class level factors. On the next step, backwards stepwise regression was used to determine which variables significantly improved the prediction. In line with Puolakanaho et al. (2007), the classification cut‐off was set to correctly identify close to 90% of poor readers. This cut‐off resulted in a sensitivity of 83.8% and a specificity of 75.1% (i.e. 83.8% of the poor readers and 75.1% of the good readers were correctly classified), giving an overall accuracy of 76.6%. The final model is shown in Table 2. PA, PK and VSTM all make a significant contribution, after school differences had been factored out.^2^

**Table 2:** Logistic regression predicting group membership

Step	Variable	Odds ratio	Confidence interval of odds ratio	*p*
1	School	2.29	1.20–4.37	.01
2	Phonological Awareness	2.92	1.20–7.07	.02
	VSTM	1.89	1.04–3.43	.04
	Print Knowledge	6.00	2.17–16.59	.01
	Rapid Naming	0.63	0.40–1.01	.053

### What deficits do poor readers show?

The percentage of each group showing deficits in each area is shown in Table 3. Children with reading difficulties are significantly more likely to show deficits in PK, PA, VSTM, vocabulary, visual search and rapid naming. They are marginally significantly more likely to show deficits in auditory processing speech rate and motor skills, but there is no significant association between reading difficulties and nonverbal reasoning or between reading difficulties and postural stability. However, there is no single deficit shown by the majority of poor readers. Only 28.3% of the group show difficulties in PA, and the most common weaknesses are in PK, VSTM and visual search.^3^

**Table 3:** Proportion of sample showing weaknesses in each area

Area of processing	Poor readers (%)	Average readers (%)	*χ* ^2^
Print knowledge	21 (44.7)	18 (8.5)	39.39, *p* < .01
Phonological awareness	13 (28.3)	27 (13.0)	6.63, *p* = .01
VSTM	19 (40.4)	25 (11.9)	22.02, *p* < .01
Sound order	11 (23.4)	28 (13.1)	3.23, *p* = .07
Motor	12 (26.1)	32 (15.0)	3.34, *p* = .07
Vocabulary	16 (33.3)	28 (12.9)	11.85, *p* < .01
Ravens	12 (25.5)	36 (16.7)	1.99, *p* = .16
Visual search	19 (40.4)	25 (11.8)	22.37, *p* < .01
Postural stability	5 (11.4)	31 (15.7)	0.54, *p* = .46
Rapid naming	15 (32.6)	28 (13.1)	10.35, *p* < .01
Speech rate	11 (25.0)	28 (13.9)	3.36, *p* = .07

### Do the data fit the multiple deficits view?

Pennington argues that children are likely to show literacy difficulties if they have multiple difficulties. Note that to examine this issue, only children with complete data can be included, which reduces the sample size to 230 (38 poor readers). The reading performance of children with a given number of impairments is shown in Table 4. There is a strong linear trend for poorer reading with increased numbers of deficits (*F*(4,230) = 14.68, *p* < .01, *η* ^2^ = .21). It appears that most children with reading difficulties show deficits in more than one area.

**Table 4:** The reading scores and proportion who are poor readers with a given number of deficits

Number of deficits	% of group who are poor readers	Mean Word Reading residualised score (*x* = 0)
0	3.3% (3/90)	0.41 (0.89)
1	15.3% (9/59)	−0.10 (0.85)
2	18.2% (6/33)	−0.25 (0.82)
3	30.4% (7/23)	−0.53 (0.70)
4–7	52.0% (13/25)	−0.82 (0.70)

### Which difficulties are associated with one another?

**Figure 2:**
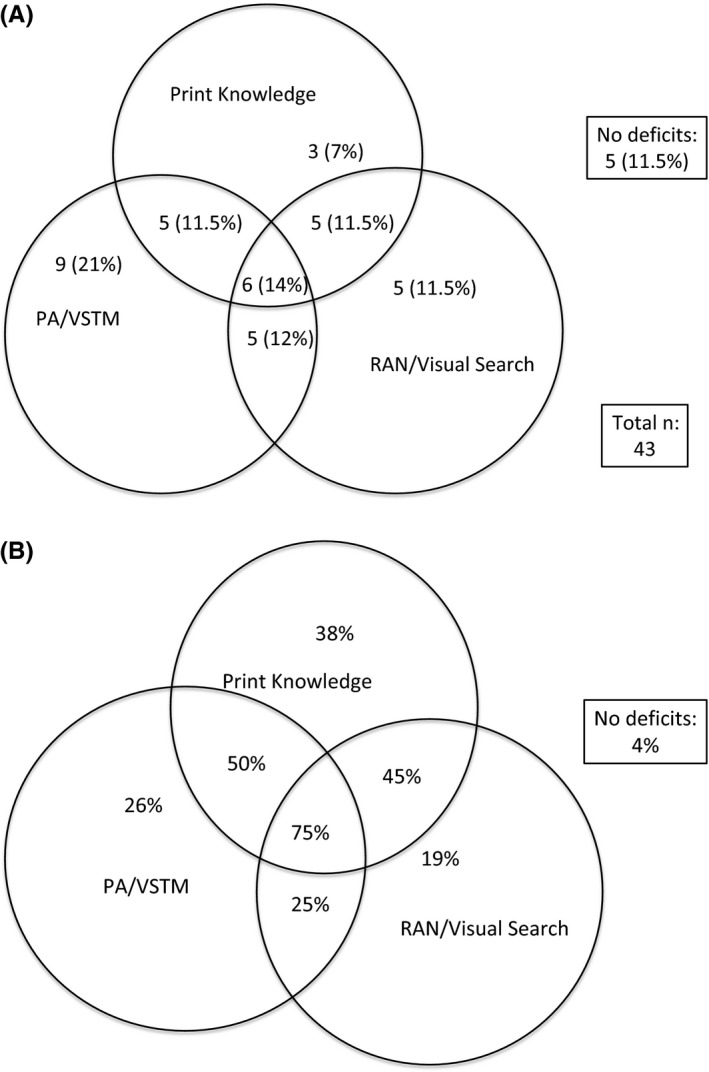
Diagrams showing the frequency of deficits in poor readers (A) and frequency of poor reading given a pattern of deficits (B)

Figure 2 shows two Venn diagrams demonstrating how the different deficits are associated. In order to simplify the data, we focussed on deficits in PK, PA, VSTM, rapid naming and visual search. Other deficits were not included either because they were not present at an increased rate in poor readers (e.g. postural stability, speech rate, motor skills, nonverbal IQ and auditory processing) or because they showed considerable overlap with other variables (vocabulary). Three different categories of deficit were considered: PA and VSTM; rapid naming and visual search; and PK. These variables were combined for both statistical and theoretical reasons: PA and VSTM are thought to be two measures of phonological processing, while visual search and rapid naming are both timed tasks involving visual scanning. In each case a child was considered to have a difficulty if they had a difficulty in either one task or the other (or both).

Figure 2A shows that only five of 43 (11.6%) poor readers showed no deficits in any area (four poor readers had missing data for one or more task). Twenty‐one of the poor readers (48.8%) showed a deficit in two or three areas, while a further 17 (39.5%) showed deficits in only one of these three areas. This indicates that poor reading is associated with a variety of different patterns of impairment at school entry, and that multiple deficits are common.

Figure 2B shows the converse analysis: given a particular pattern of deficits, how likely is it that a child will develop reading difficulties? Around 4% of children with no deficits in any area at school entry show reading difficulties later, while the majority of children with deficits in all three areas develop difficulties.

## Discussion

We examined the patterns of performance associated with later reading difficulties in an unselected sample of children at school entry. The children who were in the PR group showed a significant group disadvantage in most of the areas tested, including PA, VSTM, PK, RAN, Motor skills, visual search, vocabulary and nonverbal IQ. A greater proportion of the PR children also showed weaknesses in each of these areas. However, there was no deficit which occurred in the majority of poor readers: less than a third of the group showed deficits in PA, and less than half showed deficits in PK. Conversely, however, only a very small proportion (five children) showed deficits in none of the areas tested. The results are in line with the view that reading difficulties can have multiple different causes, and that no single cause is either necessary or sufficient for dyslexia.

The measures taken allowed us to predict future poor readers with a reasonable degree of accuracy (just over 75% of the sample were correctly classified). This level is very similar to the overall levels of accuracy achieved by Elbro et al. (1998) and Puolakanaho et al. (2007), which is impressive given that the current study used an unselected sample, rather than high risk and low risk groups. It is likely that knowledge of familial history of literacy difficulties would increase the accuracy of these predictions. In addition, these children were in their first weeks of formal schooling and prereaders at the point of testing, meaning that reading skills had not yet influenced performance on the tasks. Despite the inclusion of the wide range of cognitive‐, motor‐ and sensory‐processing tasks, the measures which provided the best predictive power in a logistic regression were PA, VSTM and PK. In a structural equation model of this dataset, PA and PK were the most important predictors of decoding and word recognition respectively (Shapiro et al., 2013), suggesting continuity between the analysis of poor readers and the sample as a whole. However, VSTM did not have a direct influence on reading in the sample as a whole. This raises the possibility that VSTM is particularly important in predicting reading difficulties. The children tested were at the earliest stages of PA and as described in Shapiro et al. (2013), at least 30% of the group were at floor on each of the PA tasks, even after excluding the least reliable measure from the analysis. The VSTM factor, which included nonword repetition, might provide a more sensitive measure of phonological processing for the lowest achieving children.

Although only three variables were significant unique predictors of reading difficulties, the poor readers showed widespread weaknesses on the tasks. As a group, performance was lower than controls on all of the measures except postural stability and speech rate. A similar pattern of results was gained when classification was based on a discrepancy criterion, confirming that deficits are linked to low reading scores and not driven by generally low cognitive abilities. This is in line with previous studies that have demonstrated weaknesses in all of these areas for reading aged dyslexic children, and demonstrates that these group level differences do exist prior to literacy tuition. However, the poor readers varied considerably in the patterns of deficits they displayed. In fact, there was no single deficit that was shown by more than half the sample of poor readers, calling into question the idea that dyslexia can be explained in terms of a single cause.

All but five of the poor readers showed deficits in rapid naming, visual search, PK, PA or VSTM, and over half of the sample showed deficits in more than one area. There are a few possible interpretations of this. It is possible that many of these deficits are epiphenomenal, as suggested by Ramus et al. (2003). That is, they occur at higher than expected levels in children with dyslexia, but do not play a causal role in the difficulties that are manifested. Alternatively, it could be that some of these difficulties represent different distal causes of more proximal difficulties. For example, it could be that rapid naming difficulties can be caused by either visual search difficulties or by difficulties in phonological retrieval (Powell, Stainthorp, Stuart, Garwood, & Quinlan, 2007). If this were the case, then one would expect proximal causes to show the greatest value in predicting outcome, but that distal causes would occur in samples of poor readers at higher than expected levels. The sample size used here unfortunately precludes this type of detailed analysis.

One of the aims of this study was to examine whether the DEST measures are accurate predictors of dyslexia. Our results parallel those of Simpson and Everatt (2005) in that Rapid Naming from the DEST, plus letter knowledge, were significant unique predictors of later literacy difficulties. In addition, deficits in auditory processing were common in the poor readers, but in our sample, phonological processing measures (phonological awareness and verbal short‐term memory) provided the most predictive validity. Measures of motor skills and balance were not closely related to later literacy.

There are some limitations to this study. Some of the measures showed floor effects at Time 1, which would have limited their predictive validity. This may have limited predictive power of the auditory and phonological processing tasks in particular. Second, some measures are included as manifest variables while others are factor scores, which reduce the error variance and allow underlying skills to be represented more accurately. The predictive power of a variable is limited by its reliability, so it is difficult to compare these directly. Further, reading difficulties have been determined by performance on a word reading measure (and, when looking at discrepancy‐defined readers, nonverbal IQ) rather than by a full diagnostic assessment, and therefore the use of the term ‘dyslexia’ is perhaps premature. In addition, we do not have detailed information on the teaching provided to individual children over the course of the study. It is possible (perhaps likely) that some children with early weaknesses received good quality intervention and therefore did not develop reading difficulties, or that some children with relatively few weaknesses have gone on to have poor reading because of low school attendance, inadequate teaching or other environmental factors. Further, it may be that multiple deficits have different effects dependent on the teaching strategies used. Further research should examine this issue in more detail. However, children were all receiving literacy tuition in line with UK government guidelines and remained in a single school over the testing period.

In conclusion, we confirmed that the best predictors of dyslexia at school entry were PK, PA, VSTM and RN. However, there was considerable individual variability among poor readers, with many different deficits linking to the disorder and most poor readers showing more than one deficit. Previous group‐based studies of children with dyslexia may have underestimated the range of individual differences in the disorder. Our findings therefore support a multiple deficits view of dyslexia.

At present the main form of intervention for children with dyslexia focuses on remediating phonological deficits, and assessment usually centres around literacy and phonological processing. Our research suggests that these approaches may not provide adequate support to these children. Broad‐based assessment and individualised intervention strategies may be a more effective approach.

**Key points**

- Classic theories of dyslexia highlight phonological processing difficulties as the cause of reading difficulties, while more recent theories highlight multiple interacting deficits, but no previous study has contrasted the full range of predictors in a sample of children covering the full range of ability.
- An unselected sample of prereading children completed a wide battery of tasks associated with reading difficulties, and their reading outcomes were measured 2–4 years later.
- There was no single deficit that was shown by more than half the sample of poor readers, calling into question the idea that dyslexia can be explained in terms of a single cause and providing support for the multiple deficits view.

## Notes

1 Group comparisons were in all cases similar for the discrepancy‐defined groups, with the exception of nonverbal IQ, which did not differ between DD children and controls (*t*(260) = −1.26, ns).

2 For the discrepancy‐defined dyslexics, phonological awareness (*p *= .02), PK (*p *< .01), rapid naming (*p *= .04) and nonverbal IQ (*p *< .01) were significant predictors of group membership, indicating that a discrepancy was more likely to be shown in those with higher nonverbal IQ, but that otherwise patterns of prediction were similar to the poor reader group.

3 In each case patterns were very similar in the discrepancy‐defined dyslexic group
